# Primary cardiac B-cell lymphoma with atrioventricular block and paroxysmal ventricular tachycardia

**DOI:** 10.1186/1749-8090-7-70

**Published:** 2012-07-18

**Authors:** Ke-Wei Chen, Ju-Hsin Chang, Su-Peng Yeh, Chiung-Ray Lu

**Affiliations:** 1 Division of Cardiology, Department of Internal Medicine, China Medical University Hospital, 2 Yuh-Der Rd, Taichung, 40447, Taiwan; 2 Departments of Anesthesia, and Pain Service and Critical Care Medicine, China Medical University Hospital, Taichung, 40402, Taiwan; 3 China Medical University Hospital, Taichung, 40402, Taiwan

**Keywords:** Primary cardiac lymphoma, AV block, PVT

## Abstract

Primary cardiac lymphoma (PCL) is very rare, and is extremely challenging to diagnose due to nonspecific symptoms. When discovered, the right atrium and ventricle are most commonly affected, while diffuse cardiac involvement is uncommon. PCL is fatal unless promptly diagnosed and treated. Herein, we present the case of a 36-year-old immunocompetent male who presented with a 5-year history of non-specific chest symptoms and was diagnosed with primary diffuse cardiac large B-cell lymphoma involving the entire heart.

## Background

Primary cardiac lymphoma (PCL) is a very rare disorder that presents with nonspecific symptoms, making diagnosis challenging [1]. The disorder accounts for approximately 1% of primary cardiac tumors and 0.5% of extranodal lymphomas, occurs three times more commonly in females than males with a median age at diagnosis of 64 years, and usually involves the right atrium and ventricle [2]. We herein present the case of a 36-year-old immunocompetent male with a 5-year history of worsening chest symptoms who was diagnosed with a primary diffuse cardiac large B-cell lymphoma.

## Case presentation

A 36-year-old immunocompetent male with history of gastroesophageal reflux presented with a 5-year history of dyspnea on exertion, chest tightness, shortness of breath, palpitations, and back pain. Physical exam revealed a heart murmur, ECG showed a Mobitz I AV block, RBBB, and right axis deviation, and chest radiograph demonstrated an enlarged heart. Laboratory studies were normal.

**Figure 1:**
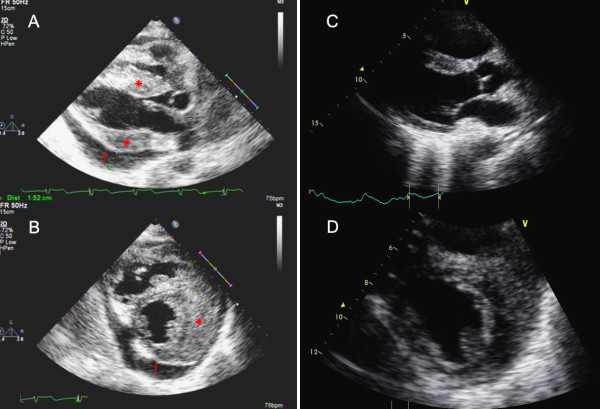
Echocardiography before treatment (left section, A: parasternal long axis and B:parasternal short axis) revealed heterogeneous infiltration involving the aortic root, and markedly increased left and right ventricular wall thickness (*). Moderate pericardial effusion (15.2 mm) was also noted (†). Six months after treatment (right section, **C:** parasternal long axis and **D:** parasternal short axis), reduced thickening of the myocardium and resolution of pericardial effusion were noted.

Transthoracic echocardiography revealed heterogeneous infiltration involving the aortic root and four chambers with marked biventricular wall thickening and moderate pericardial effusion (Figure 1A, B). The infiltrated myocardium stiffened the heart and significantly impaired systolic and diastolic functions. Computed tomography (CT) revealed a diffuse soft tissue density wrapping around the great vessels.

**Figure 2:**
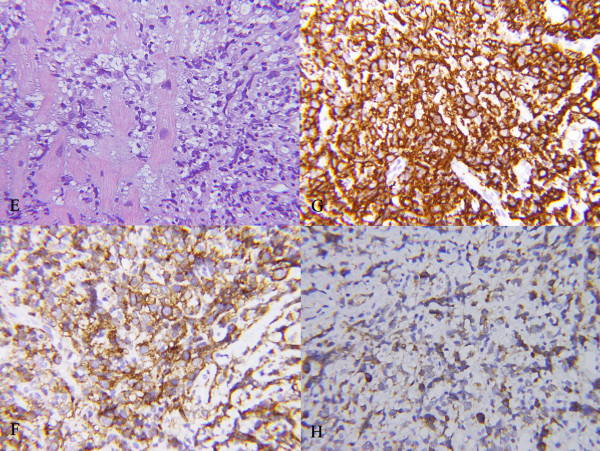
**Histological and immunohistochemical staining of the endomyocardial biopsy prior to treatment revealed atypical, intermediate to large-sized lymphoid cell infiltration in myocardium; E: Hematoxylin and eosin staining (magnification at 400×)**. **F:** anti-CD19 staining (magnification at 400×). **G:** anti-CD20 staining (magnification at 400×). **H:** anti-CD79 staining (magnification at 400×).

The patient was admitted and subsequently underwent endomyocardial biopsy prior to initiating treatment. Immunohistopathology showed infiltration of the myocardial fibers by atypical intermediate to large-sized lymphoid cell (Figure 2) that were positive for CD20, CD10, CD79, CD19, and LCA. Bone marrow biopsy revealed normocellular bone marrow, without evidence of lymphoma infiltrate. The final diagnosis was primary diffuse cardiac large B-cell lymphoma.

The patient subsequently received chemotherapy. The 1^st^ cycle consisted of cyclophosphamide/vincristine/prednisolone (COP), omitting doxorubicin due to concern of heart toxicity given the heart block. Doxorubicin was added for the 2^nd^ cycle (CHOP), and rituximab was added for the 3^rd^ through 7^th^ cycles (R-CHOP). CT showed diminished cardiac size after the first cycle. After the 2^nd^ cycle, the patient developed ventricular tachycardia and received synchronized cardioversion with resultant 1^st^ degree AV block. He then experienced paroxysmal ventricular tachycardia after the 4^th^ cycle that responded to cardioversion.

The patient responded well to chemotherapy. ECG 6 months after treatment showed normal sinus rhythm. Follow-up echocardiogram demonstrated adequate global systolic and diastolic functions, with significant reduction of myocardial thickness and resolution of pericardial effusion (Figure 1C, D).

## Discussion

The incidence of primary cardiac tumors is approximately 0.02%, and PCLs account for approximately 1% of primary cardiac tumors and the majority is diffuse large B-cell lymphoma [3]. A recent review of the literature by Miguel and Bestetti [4] for reports pertaining to the “clinical aspects, diagnosis, clinical course, or treatment” of PCL found 61 articles. Based on their review, the authors reported that PCL occurs more frequently in immunocompromised patients and those taking immunosuppressant drugs, cardiac transplant recipients, and predominantly male AIDS patients. In addition, PCL usually manifests after the fifth decades of life, and usually affects the right side of the heart. Though symptoms are non-specific and vary with the locations in the hearts affected, the most common clinical manifestations are pericardial effusion, heart failure, and AV-block [4]. Diagnosis of PCL is often delayed due to non-specific symptoms, and the prognosis is poor with a reported median survival of 7 months after initiation of treatment [5,6], though improved survival has been seen with the addition of rituximab to chemotherapy regimens [4].

The initial symptoms of PCL are non-specific and can be suggestive of many disorders including heart failure, infection, arrhythmias, and emboli [1]. Though PCL is typically seen in older patients and is associated with an immunocompromised state [1,2], our case illustrates that the condition can occur in young, otherwise healthy individuals. Interestingly, our patient presented with diffuse cardiac infiltration involving the entire heart, rather than the more commonly reported presentation involving single site such as the right atrium/ventricle [1,2].

Imaging modalities including echocardiography, CT, and magnetic resonance imaging (MRI) are useful for illustrating the cardiac involvement in PCL; however, histopathological examination of biopsied or surgical specimen is needed for diagnosis [1]. While in the past PCL was almost universally fatal, advances in imaging and anthracycline-based chemotherapy have markedly improved survival, though the median survival remains low [2].

Our patient presented with Mobitz I AV block and RBBB and developed several arrhythmias during treatment. The myocardium infiltrated by lymphoma cells becomes heterogeneous substrate, which is prone to develop cardiac arrhythmias or conduction disorders [7]. Special attention should be given to high-grade AV blocks or fatal arrhythmias during treatment of PCL [8]. Unfortunately, indications for placement of pacemaker or implantable cardioverter defibrillator in patients with cardiac lymphomas are not straightforward [8]. Given the high mortality of PCL, and resolution of conduction disorders and arrhythmias with treatment, device placement is not likely warranted in most cases. Though rare, cardiac rupture can occur with diffuse cardiac involvement and should be monitored [9].

## Conclusions

In summary, PCL can occur in young healthy individuals and should be considered in the differential diagnosis when treatments for more common conditions fail. Heart block and arrhythmias in PCL with diffuse cardiac involvement can be severe, and warrant additional vigilance during management.

## Consent

Written informed consent was obtained from the patient for publication of this report and any accompanying images.

## Competing interests

The authors declare that they have no competing interests

## Authors’ contribution

KWC drafted the manuscript, analysed and interpreted the echocardiography. JHC participated in the sequence alignment and drafted the manuscript. SPY participated in the sequence alignment and drafted the manuscript, analysed and interpreted the pathologic image. CRL participated in the sequence alignment and drafted the manuscript, analysed and interpreted the echocardiography. All authors read and approved the final manuscript.
